# External and internal training load comparison between sided-game drills in professional soccer

**DOI:** 10.3389/fspor.2023.1150461

**Published:** 2023-04-04

**Authors:** Marco Beato, Kevin L de Keijzer, Andrew J Costin

**Affiliations:** ^1^ School of Health and Sports Sciences, University of Suffolk, Ipswich, United Kingdom; ^2^ Department of Sport Science, Ipswich Town FC, Ipswich, United Kingdom

**Keywords:** football, team sports, performance, GPS, monitoring

## Abstract

This study aims to quantify and compare the external and internal training load demands of sided-game drills in professional team players during the competitive season. Twenty-four male professional soccer players of the same club were enrolled in this study. Drills were categorized as large-sided games (LSG): 10vs10 (84 × 60 m or 72 × 60 m), Hexagon possession 9vs9 + 3 (36 × 48 m), Possession gate 8vs8 + 2 (36 × 44 m), Possession 7vs7 + 3 (30 × 32 m) or as Small-sided games (SSG): 6vs6 (48 × 42 m), and Possession 6vs4 (30 × 60 m). A total of 7 drills and 279 individual data points were included in this analysis. Distance covered, high-speed running (HSR), and sprinting distance were all calculated in meters per minute (m.min^−1^) while total accelerations (>3 m.s^−2^) and total decelerations (− < 3 m.s^−2^) were calculated in number of actions per minute (n.min^−1^). All external load was measured with global navigation satellite systems (GNSS) STATSports Apex units. Players’ internal load was quantified using their rating of perceived exertion (RPE). We found that distance covered (*p* < 0.01, *large*), HSR (*p* < 0.01, *large*), and sprinting distance (*p* < 0.01, *large*) changed between drills (e.g., greater in LSG formats), acceleration (*p* < 0.01, *large*) and deceleration (*p* < 0.01, *large*) demands were greater in smaller formats (e.g., SSG 6vs6, and Possession 6vs4), while RPE was lower in the Possession gate 8vs8 + 2 format (*p* < 0.01, *large*). This study found that sided-games can replicate and sometimes exceed some match-specific intensity parameters, however, HSR and sprinting were consistently lower compared to official matches.

## Introduction

Soccer requires players to have a high level of fitness to consistently execute the technical and tactical demands of the game (1, 2). The appropriate combination of these three factors in the training process plays a paramount role in short- and long-term preparation for competition (3, 4). Sided-games are a common form of training used to enhance performance and prepare players in professional soccer (5, 6). Coaches use sided-game drills that vary in pitch size, rules and in number of players to develop the physical (*e.g*., aerobic fitness, speed), psychological, technical and tactical (*e.g.,* possession skills, pressing) skills needed in soccer (7). Although sided-games are commonplace in training, the application of such drills may insufficiently replicate the physical demands of the game (8).

It is important for coaches and sport scientists to track the external and internal demands of training to enhance physical capacity (3, 9). External load is commonly monitored using global navigation satellite system (GNSS) units across elite and semi-professional soccer teams (10–12). GNSS units acquire and track multiple satellite systems (e.g., Global Positioning System, GLONASS) and have evolved to provide practitioners with a more accurate and holistic understanding of the demands placed upon soccer players (10, 13). In addition to the monitoring of distance and velocity data, GNSS units have an integrated triaxial accelerometer (e.g., acquisition frequency usually of 100 Hz) that allows for evaluation of additional accelerometer-based parameters (14). Specifically, practitioners often assess acceleration and deceleration efforts (considered above a threshold of >3 m.s^−2^ and <−3 m.s^−2^, respectively) during training and competition (15). The analysis of external training load can be implemented alongside the monitoring of internal training load parameters such as heart rate, blood lactate, and the player's rating of perceived exertion (RPE) (16, 17). However, the daily recording of heart rate and blood lactate remains challenging, while the use of RPE is non-invasive, cheaper, and easier to implement (16). The construct validity of RPE has been reported in several studies and was found to be strongly correlated with heart rate (*r* = 0.74) and blood lactate (*r* = 0.83) during aerobic exercise (4, 18). For these reasons, RPE can give an overall indication of a players' internal load (18).

The monitoring of both external (e.g., distance, high-speed running [HSR], accelerations, decelerations) and internal load (e.g., RPE) parameters and the consequent manipulations of training variables (sided-games rules and spaces) play a key role in players' fitness development throughout the season and for players' readiness for competition (3, 17, 19). Sided-games in soccer have received a lot of scientific interest and support throughout the years because of their ability to achieve adequate internal load demands (*i.e.,* around 85%–90% of maximum heart rate) and stimulate aerobic fitness amongst soccer players (5–7). Additionally, sided games are attractive to coaches because they offer a large variety of challenges and enable players to train certain technical aspects and tactical principles in greater detail. Coaches also manipulate the objectives of games (possession or goal-oriented) and vary rules (goal size, presence of neutral players, or numerical player overloads) to achieve different psychophysical (*e.g.*, RPE, heart rate) and mechanical load (*e.g.,* accelerations) objectives and stimuli (7, 15). However, a recent systematic review by Dello Iacono et al. (8), reported that sided-games (ranging from small to large) are inadequate for training the higher speed demands of the game. Specifically, sided-games consistently offered a lower dose of high-speed running and sprinting distance (per unit of time) compared to official matches (8).

Further investigation is necessary to better understand whether sided-games formats (with different rules and objectives) elicit a similar training load as well as it is needed to verify if the sided games used with professional players in an ecological context can replicate the physical demands of regular matches. Therefore, the aim of this study was to quantify and compare the external and internal training load demands of sided-game drills in professional team players during the official season. We aimed to verify, first, if different sided-game formats can actually offer different physical stimuli and second, if the intensity (per unit of time) reported for the external load metrics recorded were adequate to stimulate players compared to the intensity reported during matches.

## Methods

### Participants

Twenty-four male professional soccer players of the same club were enrolled in this study (age = 27 ± 9 years old and body mass = 79 ± 15 kg). The inclusion criteria were the absence of illness and injuries and regular participation in soccer competition. Goalkeepers (GKs) were excluded in this study and only outfield players match data were evaluated. The sample size power was evaluated using G*power (Düsseldorf, Germany) for an ANOVA fixed effects, one way and results indicated that a total of 119 individual data points would be required to detect a *moderate* effect (*f *= 0.35) with 80% power and an alpha of 5%. The actual sample size of this study was of 279 individual data points, with a real power of >95%, which reduced the likelihood of type 2 error (false negative) (20).

External training load data was recorded as part of the normal monitoring routine of the club and was analyzed *a posteriori*. The Ethics Committee of the University of Suffolk (Ipswich, UK) approved this study (project code: RETHS22/016). Informed consent to take part in this research was signed by the club. All procedures were conducted according to the Declaration of Helsinki for human studies.

### Experimental design

Drills were categorized as A) Large-sided games (LSG) 10vs10 (84 × 60 m), B) LSG 10vs10 (72 × 60 m), C) Hexagon possession 9vs9 + 3 (36 × 48 m), D) Possession gate 8vs8 + 2 (36 × 44 m), E) Possession 7vs7 + 3 (30 × 32 m), F) Small-sided games (SSG) 6vs6 (48 × 42 m), G) Possession 6vs4 (30 × 60 m). Only players that played for the full duration of the drill were included in this analysis. A total of 7 drills and 279 individual data points were included in this analysis. Offside rule was present during LSG formats only. No restriction on player's ball touches was applied for any sided-game drills. Additional balls were available around the pitches and were used to replace a ball that went out of the pitch—this was to allow the maintenance of intensity.

#### Sided-game drills description

LSG 10vs10 (84 × 60 m) and B) LSG 10vs10 (72 × 60 m) are sided-games that simulate a soccer match (with the same rules), involving regular goals and GKs, but in restricted space compared to a regular match, 229 m^2^ and 196.4 m^2^, respectively. The duration of these drills ranged from 7 to 10 min.
Hexagon possession 9vs9 + 3 (36 × 48 m) is a possession drill with 3 neutral players free to move within a hexagon shaped pitch. The aim of the drill is to maintain possession of the ball for as long as possible and score “goals” by completing 6 passes. The opposition team are instructed to win the ball and instantly switch focus to maintain possession and score “goals” by completing 6 passes. The relative space size of the drill was 82 m^2^. The duration of this drill was 8 min.
Possession gate 8vs8 + 2 (36 × 44 m) is a possession drill with 2 neutral players free to move on the pitch. The aim of the activity is to pass a ball through one of 3 gates (*i.e.*, small goals) where a teammate must receive the pass. The opposition team are instructed to win the ball and then instantly switch focus to scoring by passing the ball through one of 3 gates. The relative space size of the drill was 88 m^2^. The duration of this drill was 7 min.
Possession 7vs7 + 3 (30 × 32 m) is a possession drill with 3 neutral players free to move within a rectangular shaped pitch. The aim of this drill is to maintain possession of the ball for as long as possible and score “goals” by completing 10 passes. The opposition team are instructed to win the ball and instantly switch focus to maintain possession for as long as possible. The relative space size of the drill was 56.5 m^2^. The duration of this drill was 7 min.
SSG 6vs6 (48 × 42 m) are sided-games that involve regular goals and GKs, but in more restricted space compared to LSGs (*i.e.,*168 m^2^). In this specific drill, the use of GKs and regular goals were used to maximize intensity. The duration of this drill ranged from 5 to 6 min.
Possession 6vs4 (30 × 60 m) is a possession drill with the aim of maintaining possession of the ball for as long as possible and scoring “goals” by completing 10 passes. The opposition team try to win the ball and then instantly switch focus to maintain possession for as long as possible. The relative space size of the drill was 180 m^2^. The duration of this drill ranged from 5 to 6 min.

#### GNSS and data recording procedure

STATSports 10 Hz GNSS units (STATSports, Northern Ireland) with integrated 100 Hz triaxial accelerometer acquire and track multiple satellite systems (*i.e*., global positioning systems, GLONASS) to provide highly accurate and reliable positional information (10). Apex units were validated for both linear and soccer-specific distances, reporting an error between 1 and 2.5% (10). The inter-units' reliability for sprints was previously reported and classified as *excellent* (intra-class correlation coefficient = 0.99), with a typical error of measurement of 1.85% for sprints ranging from 5 to 30 m (21).

Before each data recording, the GNSS Apex units were turned on about 15 min before the beginning. These units reported the quality of the signals that ranged between 17 and 21 satellites, which is in line with previous literature (21). All data recorded by the Apex units were downloaded and elaborated by STATSports software (Apex version Sonra v4.4.17) before being exported as a CSV. file for further analysis.

#### External and internal load variables

Distance covered, HSR distance (over 5.5 m·s^−1^ or 19.8 km^.^h^−1^), and sprinting distance (over 7.0 m·s^−1^ or 25.2 km.h^−1^) were analyzed in meters per minute (m^.^min^−1^) (19). Total accelerations (>3 m^.^s^−2^) and total decelerations (− < 3 m.s^−2^) were analyzed as number of actions per minute (n.min^−1^) (3, 15, 22). All external load metrics were reported as frequency per minute to decrease the difference of training (time) exposure. Players' internal load was quantified using their rate of perceived exertion (RPE) (Borg's CR10 scale) and expressed in arbitrary units (AU) (16). The construct validity of this scale was previously reported such as RPE was strongly correlated with heart rate (*r* = 0.74, *p* < 0.001) and blood lactate (*r* = 0.83, *p* < 0.001) during aerobic exercise (18).

### Statistical analyses

Descriptive statistics are reported as mean ± standard deviation (SD). A Shapiro-Wilk test was used to check the assumption that the data conforms to a normal distribution. An analysis of variance (ANOVA) test was used to assess if significant differences exist between drills across several dependent variables. Effect sizes were reported using the eta squared (*η*^2^) that express the amount of variance accounted for by one or more independent variables. *η*^2^ was interpreted as >0.01 *small*, >0.06 *medium* and >0.14 *large* effect. If data were not normally distributed, a Kruskal-Wallis Test (non-parametric ANOVA) was performed. A homogeneity (equal variances across samples) test was performed using the Levene's test, and if a violation was found, the Brown-Forsythe correction was applied. When significant differences were found in the ANOVA, *post hoc* analysis was performed using Bonferroni corrections. Estimates of 95% confidence intervals (CIs) were calculated and reported in the figures. Effect sizes were interpreted using Cohen's *d* principle as follows *trivial *< 0.2, *small* 0.2–0.6, *moderate* 0.6–1.2, *large* 1.2–2.0, *very large* > 2.0 (23). Unless otherwise stated significance was set at *p* < 0.05 for all tests. Statistical analyses were performed in JASP (JASP Version 0.16.13. Amsterdam, Netherlands).

## Results

Summary of the comparison between training load parameters during different drills is reported in Table 1.

**Table 1:** Comparison between training load parameters during different drills.

Variable	*F*-value	*P*-value	Effect size (*η*^2^)	Qualitative interpretation
Distance per minute (m.min^−1^)	33.5	<0.001	0.426	Large
HSR per minute (m.min^−1^)	19.2	<0.001	0.298	Large
Sprinting per minute (m.min^−1^)	6.6	<0.001	0.127	Large
Accelerations per minute (n.min^−1^)	23.3	<0.001	0.340	Large
Decelerations per minute (n.min^−1^)	22.36	<0.001	0.331	Large
RPE (AU)	76.2	<0.001	0.628	Large

High-speed running (HSR), RPE = rating of perceived exertion. Eta squared (*η*^2^) express the amount of variance accounted for by one or more independent variables. *η*^2^ was interpreted as >0.01 *small*, >0.06 *medium* and >0.14 *large* effect. Unless otherwise stated significance was set at *p* < 0.05 for all tests.

**Figure 1:**
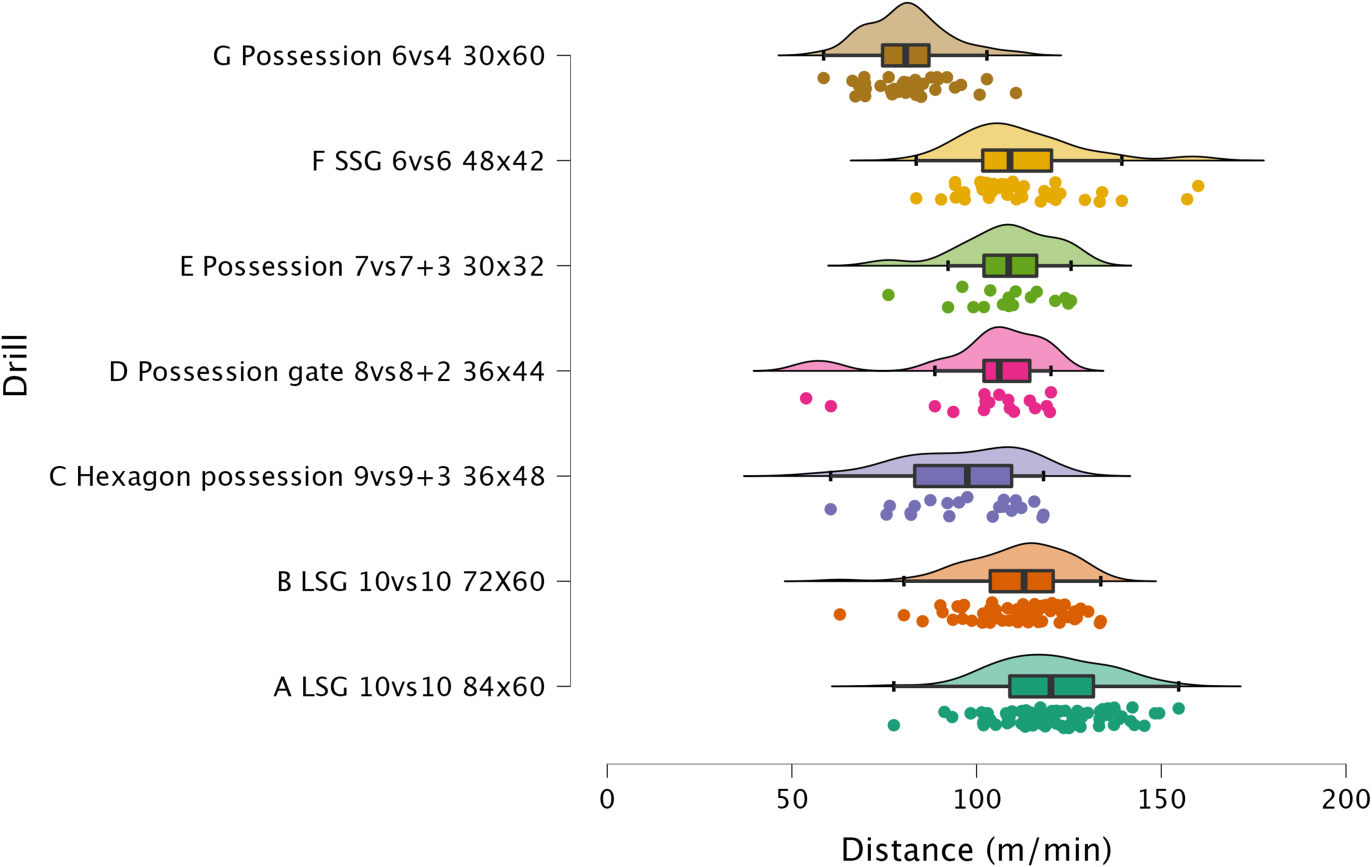
Comparison of distance per minute between drills. The data distributions show, mean, standard deviation, 95% confidence intervals, and individual data points.

**Figure 2:**
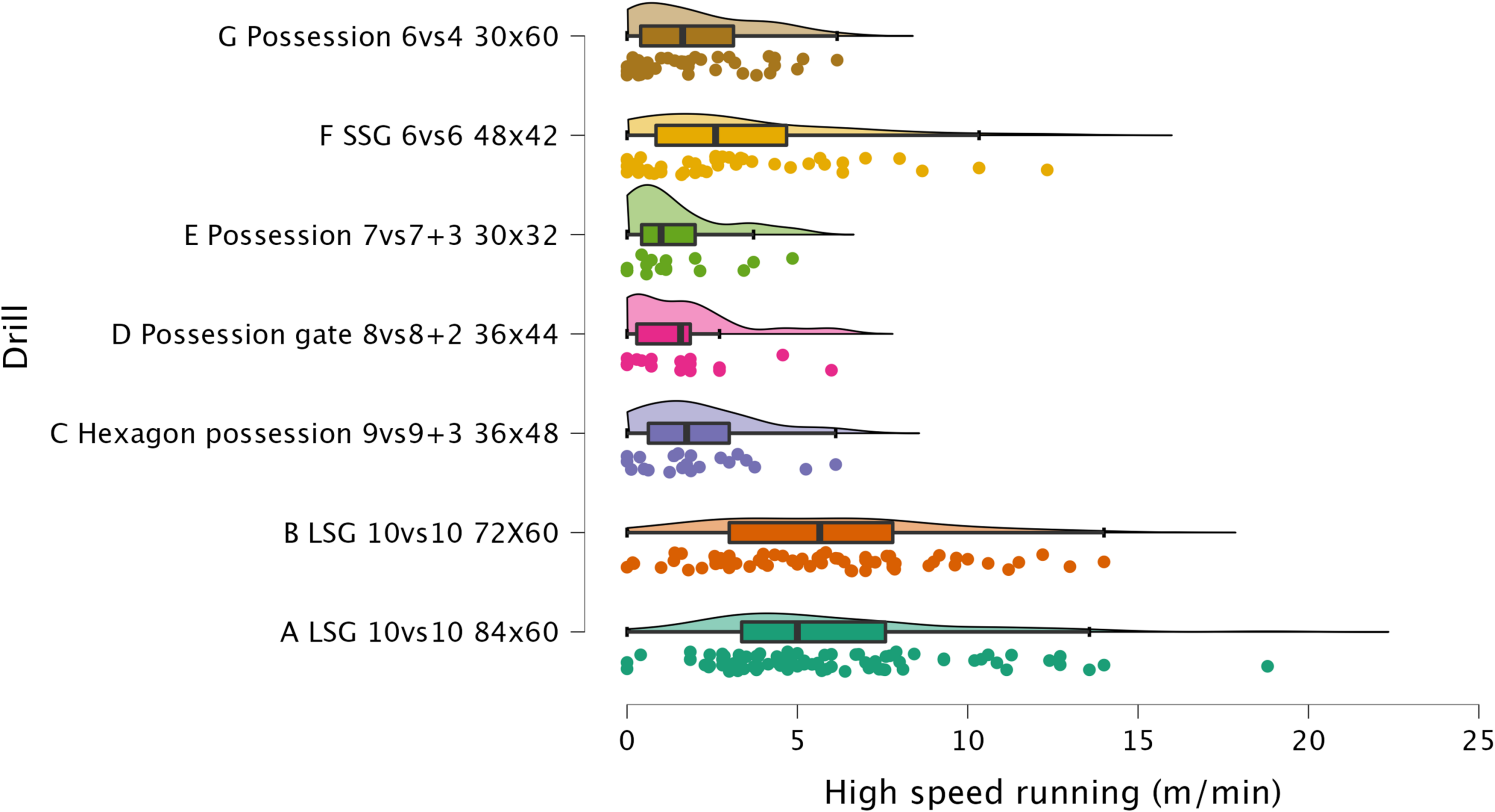
Comparison of HSR per minute between drills. The data distributions show, mean, standard deviation, 95% confidence intervals, and individual data points.

**Figure 3:**
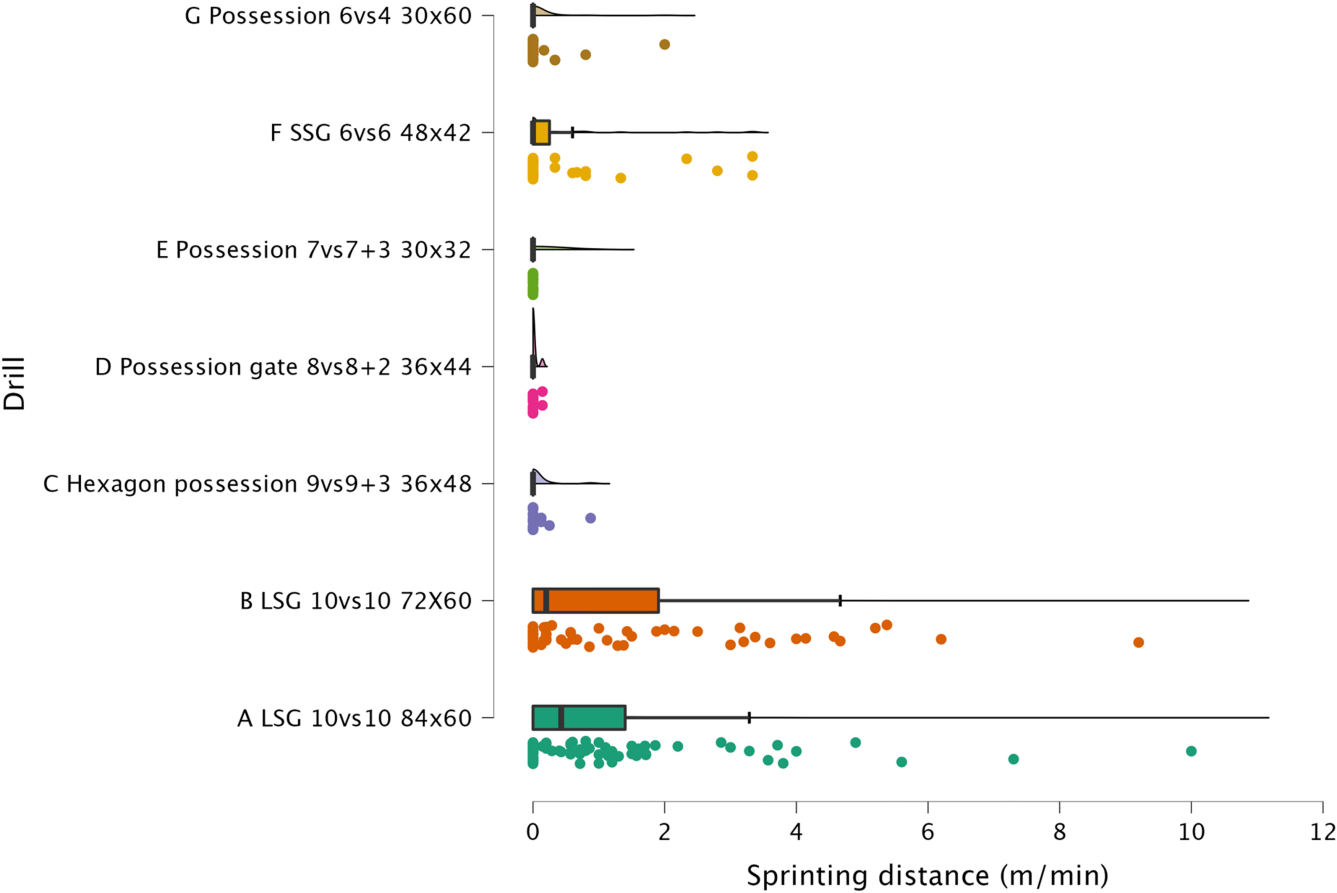
Comparison of sprinting distance per minute between drills. The data distributions show, mean, standard deviation, 95% confidence intervals, and individual data points.

**Figure 4:**
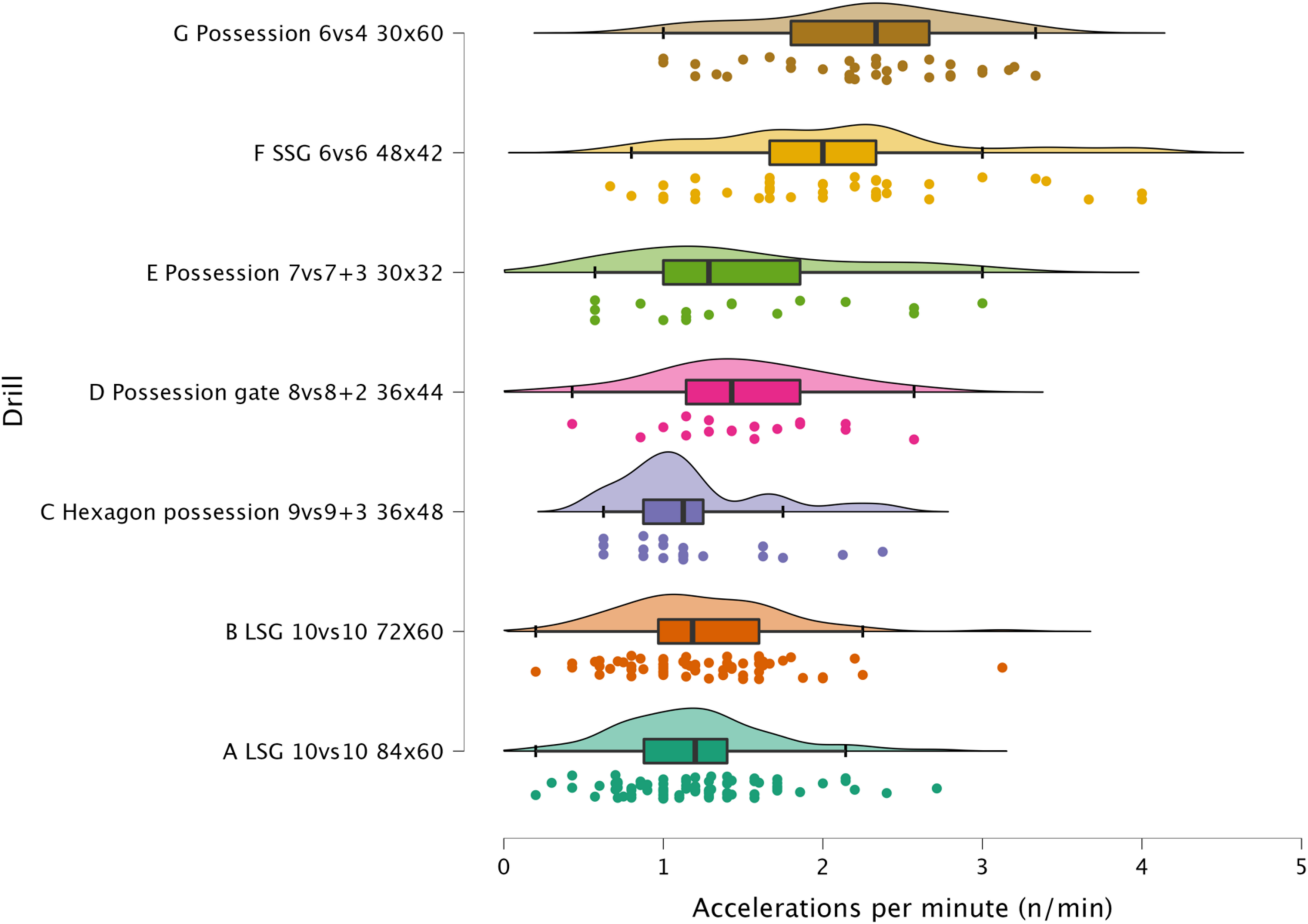
Comparison of accelerations per minute between drills. The data distributions show, mean, standard deviation, 95% confidence intervals, and individual data points.

**Figure 5:**
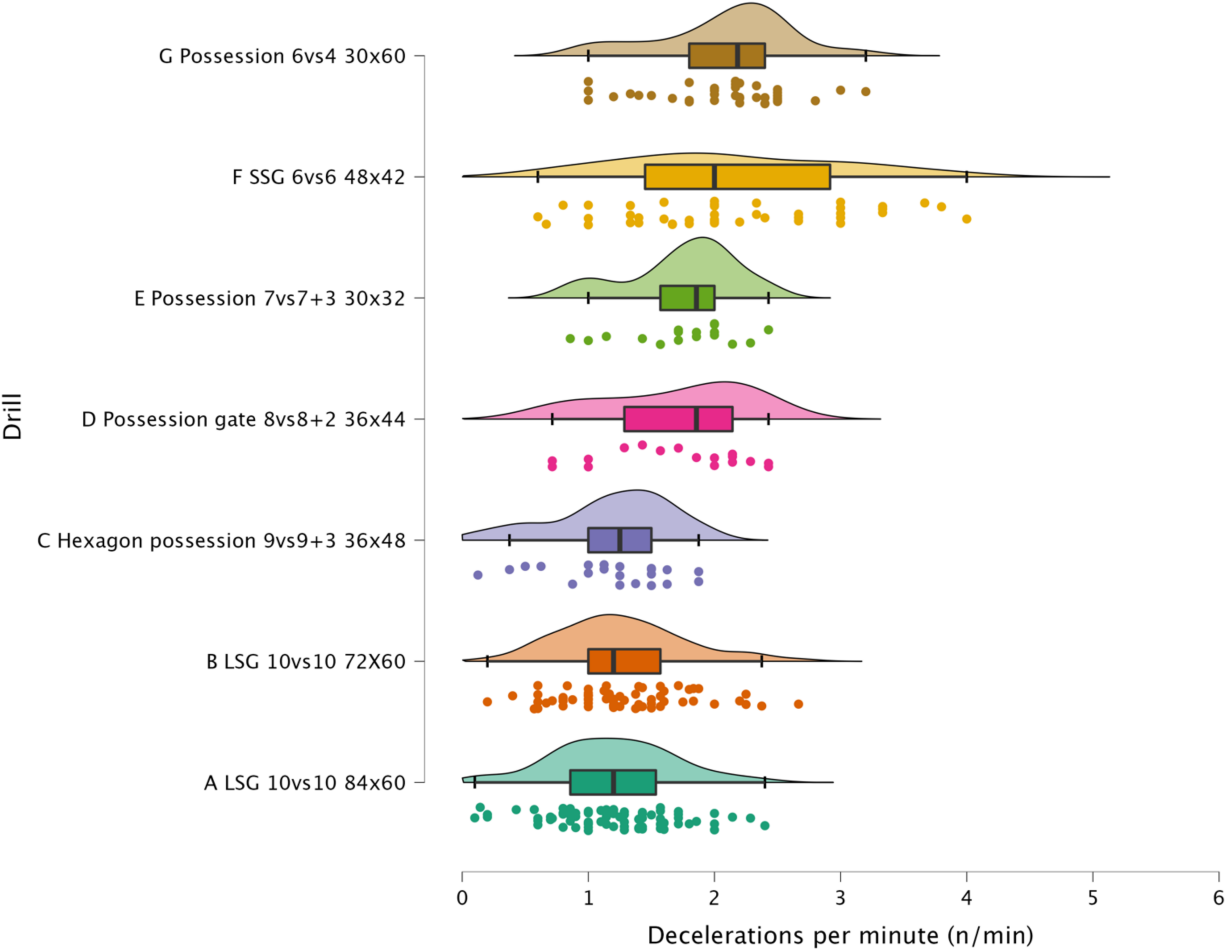
Comparison of decelerations per minute between drills. The data distributions show, mean, standard deviation, 95% confidence intervals, and individual data points.

**Figure 6:**
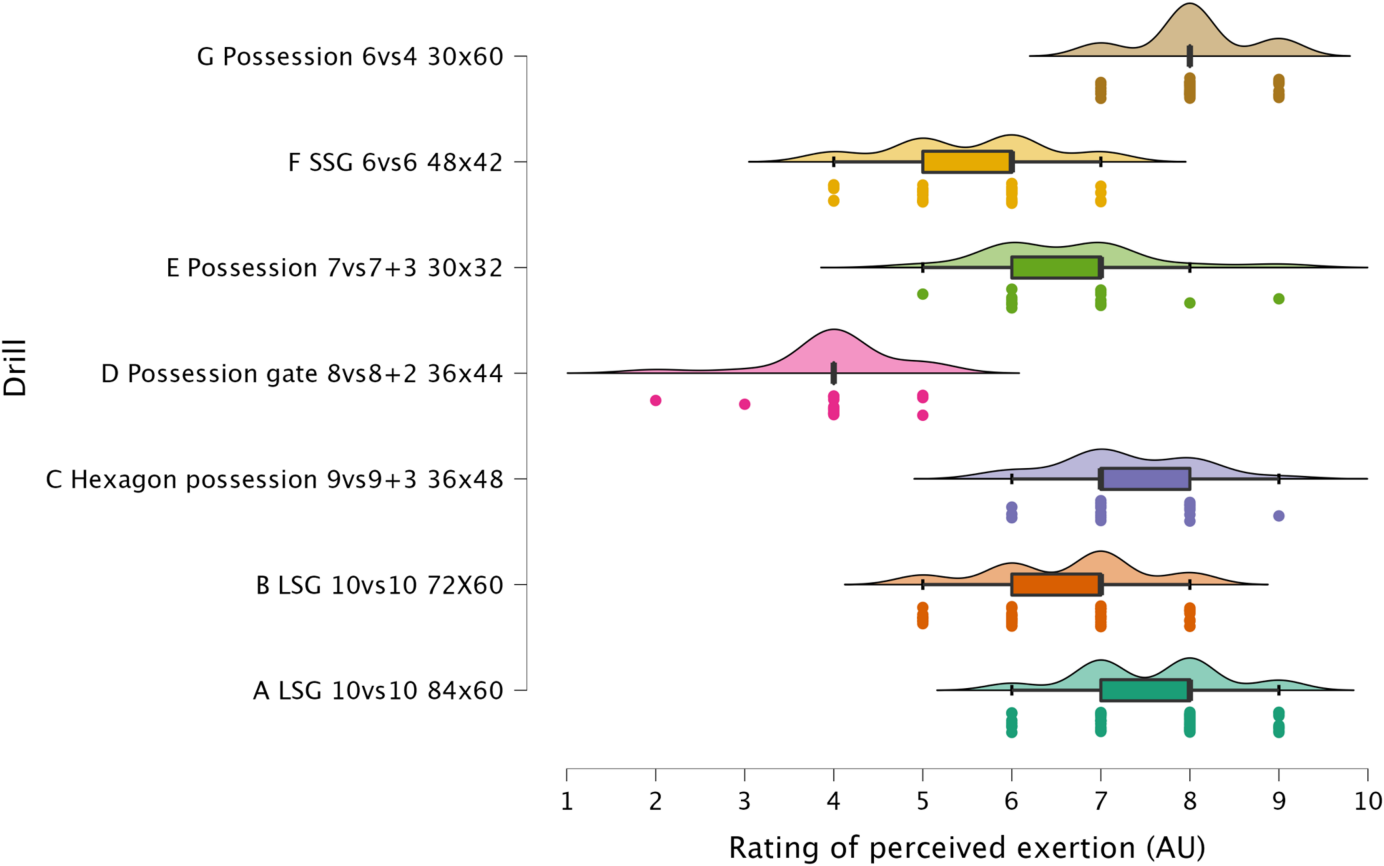
Comparison of RPE between drills. The data distributions show, mean, standard deviation, 95% confidence intervals, and individual data points.

The results of ANOVA for each external load variable are reported in the following figures: distance per minute in Figure 1, HSR per minute in Figure 2, sprinting distance per minute in Figure 3, accelerations per minute in Figure 4, decelerations per minute in Figure 5, RPE in Figure 6.

Post hoc analysis reporting delta difference, *p*-values with Bonferroni corrections and Cohen's *d* effect size for each drill and training load metric was reported in the supplementary material.

### Discussion

The aim of this study was to quantify and compare the external and internal training load demands of sided-game drills in professional team players during the official season. In this study we analyzed a variety of formats: LSG 10vs10, Hexagon possession 9vs9 + 3, Possession gate 8vs8 + 2, Possession 7vs7 + 3, SSG 6vs6, and Possession 6vs4. The first aim was to verify if different sided-game formats can offer different physical stimuli. We found that distance and HSR were greater in large-sided game (LSG) formats (LSG 10vs10) while acceleration and deceleration demands were greater in small-sided game (SSG) formats (SSG 6vs6 and Possession 6vs4). Interestingly, RPE was lower in the Possession gate 8vs8 + 2 format in comparison to all the other formats. These findings support the need for monitoring of training load during different sided-game formats implemented due to the large variation between drills in external and internal training load. The second aim of this study was to compare if the intensity of the external load metrics recorded were adequate to stimulate players compared to the intensity reported during matches. Although we found that sided-games can indeed replicate and occasionally exceed some intensity parameters, HSR and sprinting were consistently lower than what is found in official matches.

#### Distance per minute

Previous research reported that sided-game formats can be adapted to offer different physical demands (*i.e.*, distance per minute) (5, 24). LSG formats are usually used to obtain higher distance covered compared to SSGs or other formats (19). In this study, LSG 10vs10 (84 × 60 m and 72 × 60 m) was reported to have an average of 120.1 m.min^−1^ and 111.1 m.min^−1^, respectively (Figure 1). In this case, a reduction in relative space size from 229 m^2^ to 196.4 m^2^ shows a significant (*p* < 0.05, d = 0.61, *moderate*) decrement in distance per minute. These values are supported by previous research that reported that sided-games ranged from 14.8 m⋅min^−1^ to 17.2 m⋅min^−1^ (8). Other formats such as Hexagon possession 9vs9 + 3 (duration = 8 min) and Possession gate 8vs8 + 2 (duration = 7 min) showed 96.8 m.min^−1^ and 101.7 m.min^−1^, which were significantly lower (*p* < 0.01, *very large*) than LSG 10vs10 84 × 60 m (duration = 10 min). The relative space of the drill was 82 m^2^ and 88 m^2^, respectively. Therefore, it seems quite clear that a reduction in relative space size reduces the distance per minute covered by players (25). A second factor that can affect distance per minute is the rules used during sided-games (7). For instance, Possession 6vs4 required players to play in an imbalanced way, specifically, the team with the ball would try to maintain its possession for as long as possible and “goals” were scored based on making 10 passes. Although the relative space size was 180 m^2^, the rules of the game did not enable the players to cover (81.3 m.min^−1^) the same distance per minute of other possessions games. For example, possession 7vs7 + 3 (56.5 m^2^) had an average of 108.3 m^.^min^−1^ (d = 1.84, *large*). These results (see, Figure 1) show that a combination of appropriate relative space size and game rules are necessary to obtain the wished distance per minute output.

#### HSR and sprinting distance

In recent years, HSR and sprinting distance have been reported as among the most important external load variables for monitoring in soccer (3). Exposure to high-speed activities has a dual aim, first, to train the players for the demands of the match, second, to decrease the probability of lower limb non-contact muscular injuries (*i.e*., hamstrings) (19, 26). In this study, we have observed that HSR and sprinting distance are greater in LSG compared to SSG (SSG = 168 m^2^) and other possession formats played on a smaller relative space size (see Figure 2 and Figure 3). LSG formats (229 m^2^ and 196.4 m^2^) enabled for greater HSR (5.9 and 5.6 m.min^−1^, respectively) while 6vs6 SSG only enabled for 3.2 m.min^−1^ of distance, which was significantly lower (*p* < 0.01, *moderate*). Significant differences were also found for sprinting distances, where players achieved 1.1 and 1.3 m.min^−1^ in the LSG formats compared to a very low sprinting distance of 0.4 m.min^−1^ during SSG formats. After a visual analysis of Figure 2, it is very clear that HSR is mainly achieved in LSG formats, while other formats such as SSG 6vs6 and Hexagon possession 9vs9 + 3 only offer lower exposures—most sided-game formats obtain trivial (<2 m.min^−1^) exposures. A similar visual analysis of Figure 3 shows very clearly that sprinting activity is mainly performed in LSG formats (although the actual distance per minute is minimal), while all the other sided-games show an average exposure lower than 1 m^.^min^−1^. Therefore, a practical recommendation for practitioners is to use formats with a relative space size > 200 m^2^ to generate HSR and sprinting distance with their players. The values found in this study are supported by a previous systematic review that found HSR ranged from 2.7 m⋅min^−1^ to 3.6 m⋅min^−1^ and sprinting distance ranged from 0.2 m⋅min^−1^ to 0.7 m⋅min^−1^ in a large sample (*n* = 104) of sided-games studies (8). It is clear from the previous research and from the results of this study that sided-games enable for a limited HSR exposure (apart from LSG, mean ranges = 5.9 and 5.6 m.min^−1^) and very limited (if not trivial) sprinting distance exposure.

#### Accelerations, decelerations and RPE

Sided-games are frequently used in soccer to generate a mechanical load in the players' lower limbs. Although this mechanical load cannot be easily quantified (22, 27), sport scientists and coaches monitor the number of accelerations and decelerations performed during soccer-specific drills (15). In Figure 3 and Figure 4 is possible to observe that all formats used in this study can provide exposure to this type of actions that could be suitable to replicate the demands of the game and so to maintain/enhance physical performance. Of the sided-games monitored in this study, it is clear that Possession 6vs4 and SSG 6vs6 are more suitable than LSG formats for achieving accelerations and deceleration demands. The acceleration and deceleration demands of the possession 6vs4 and SSG 6v6 ranged between 2.1 and 2.2 n.min^−1^, while only 1.2 accelerations or decelerations n^.^min^−1^ were performed with LSG formats (Figures 4 & 5). The exposure to acceleration and deceleration efforts was significantly lower during LSG formats compared to Possession 6vs4 (*p* < 0.01, *moderate*). In addition to the use of external load parameters, practitioners can assess the players' internal load using an RPE scale, which is cheap and easy to implement (28, 29). RPE enables for a subjective quantification of the overall load that the players have perceived during the sided-games (7, 16). RPE correlates with internal load parameters (heart rate and blood lactate) (18) and has also been found to be sensitive to changes in acceleration intensity (22). In Figure 6, it is possible to evaluate the RPE of the sided-games assessed in this study; we can see that Possession gate 8vs8 + 2 shows the lowest score (RPE = 4.00 au) amongst all drills, while Possession 6vs4 and LSG 10vs10 (84 × 60 m) show the highest scores 8.0 and 7.5 au, respectively. The difference between Possession gate 8vs8 + 2 and the other two formats is *very large* and significant (*p* < 0.01), while the difference between the other drills ranges from *small* to *very large*. Although these results are of interest, partitioners need to be aware that RPE gives an indication of the overall perceived load, but it is not clear exactly what this score is composed of. Specifically, it is possible to see that Possession 6vs4 and LSG 10vs10 (84 × 60 m) have no significantly different scores (*p* = 0.073), but the formats characteristics and the external load parameters recorded are very different between these drills. Possession 6vs4 is played on a relative space size of 180 m^2^, while a LSG 10vs10 (84 × 60 m) has relative space size of 229 m^2^, the first is a possession game (with specific tactical aims), while the second is a goal-oriented format with different tactical aims. The HSR in Possession 6vs4 format is 1.9 m.min^−1^ vs. 5.9 m.min^−1^ of LSG 10vs10 (84 × 60 m), moreover, the number of accelerations were 2.2 per minute vs. 1.2 per minute. Therefore, professionals can use RPE to evaluate the overall perceived load of players during sided-games, however, some important considerations for its use and interpretation need to be made. The players' perceived exertion values can derive from different factors and cannot be easily interpreted when analyzed in isolation. For instance, sided-games with very different characteristics and demands (accelerations or HSR) could give very similar RPE scores, however, the external load parameters and the tactical characteristics of the drills can be very different (and consequently, the real physical stimulus). Therefore, we suggest practitioners avoid focusing only on the use of RPE but to integrate external and internal load parameters in their monitoring system. These suggestions are supported by previous research that found very similar RPE scores during soccer-specific training protocols (30), although the accelerations and HSR demands of these formats were significantly different among them. The data reported in the literature (8, 12, 30), in addition to what found in the current research suggest the necessity for practitioners of assessing external load parameters in soccer to have a more complete understanding of players' training load.

#### Sided-games vs. matches

The sided-games monitored in this study should be compared with the intensity reported during official matches in order to understand if they can adequately train players for the intensity of the game. Previous research that analyzed players of a similar level (EFL League 1) reported that they covered a distance of 105.6 m.min^−1^ (31). The drills analyzed in this study had scores all above this intensity except for Hexagon possession 9vs9 + 3 (96.8 m.min^−1^), Possession gate 8vs8 + 2 (101.7 m.min^−1^), and Possession 6vs4 (81.3 m.min^−1^). Therefore, if practitioners aim to replicate the distance per minute of official matches, they should select their drills accordingly (see Figure 1). However, practitioners should be aware that higher level football players (Dutch Eredivisie) reported higher distances 121.4 m^.^min^−1^ (32), therefore, the intensity found in this study should be reassessed when different players are used. Regarding HSR and sprinting distance, intensities of 7.0 m.min^−1^ and 1.5 m.min^−1^ were reported during official games, respectively (31). Observing the intensities indicated in Figure 2 and Figure 3, it is possible to report that with the exception of the LSG formats, all the sided-games analyzed in this study offer an intensity that is much lower than what is reported during competitive matches (31). This is a critical point because sided-games are extremely popular training formats in soccer (7) but generally fail to fully prepare players for the high-speed demands of the game (8). Practitioners should therefore add other drills to their training routine (*i.e.,* ball-based circuit drills) (30) and linear sprinting exercises (without the ball) to prepare their players for competition (19, 33). Sided-games are also used to generate a mechanical load in soccer players, mainly because they offer exposure to acceleration and deceleration actions (15, 34). The physiological benefits of acceleration and deceleration activities (*i.e.*, short-shuttle runs) were well described in previous papers (35, 36). From a match perspective, players generally perform around 60–80 accelerations and decelerations per match (32, 37, 38), which mean around 0.6 and 0.9 actions per minute. Figure 3 and Figure 4 highlight that acceleration and deceleration demands during sided-games ranged from 1.2 to 2.2 and 1.2 to 2.1 n.min^−1^, respectively. Our findings confirm that these drills can offer an adequate mechanical stimulus to prepare players for match demands. Practitioners can therefore use and manipulate the drill formats in the current study to generate the adequate mechanical load for their players as reported in the literature (15).

#### Limitations and future directions

This study is not without limitations, first, the players monitored in this study are professional athletes playing in EFL League 1, therefore, the intensity found could be different if higher- or lower-level players would perform the same sided-games. Therefore, practitioners of different clubs should verify these intensities with their players if using the same drills suggested here. Secondly, this study enrolled a sample of male professional soccer players, therefore these data cannot be easily used on female soccer populations. Recent research reported that more information and in particular more original studies are needed to increase the knowledge about female soccer (39), specifically, the number of articles are not comparable to current research output levels in male football. Thirdly, this study used a specific GNSS technology to monitor the external training load of the drills (10). Each technology has different accuracy and in particular, for the accelerations and decelerations, differences in filtering and acquisition frequency can make it difficult to compare outcomes amongst studies (15, 40).

## Conclusions

This study found that the external and internal training load demands vary among sided-game drills in professional team players. Sided-game formats should be selected based on the coaches' technical and tactical aims but also consider the physical outcomes that they want to obtain. Match intensities can be trained using LSG 10vs10, SSG 6vs6 and Possession 7vs7 + 3 formats for some performance parameters (*e.g.*, distance per minute, accelerations, decelerations). LSG 10vs10 are the most suitable formats to achieve HSR and sprinting distance objectives, although the intensities recorded are lower than what was observed during regular matches. Practitioners should therefore also use other training methods to compensate for the external load recorded during sided-games such as ball-based circuits and linear sprinting drills. The acceleration and deceleration load can be comfortably achieved with several sided-games and in particular with SSG 6vs6 and Possession 6vs4 formats which offer a higher frequency of acceleration and deceleration actions per minute. Finally, RPE can be used as a subjective measure of perceived load, but practitioners need to be aware that sided-games with very different characteristics and load demands (accelerations and HSR) could obtain very similar RPE scores. Therefore, we suggest practitioners avoid focusing only on the use of RPE but integrate external and internal load parameters comprehensively within their monitoring system.

## Data Availability

The raw data supporting the conclusions of this article will be made available by the authors, without undue reservation.

## References

[1] Hoff J Wisløff U Engen LC Kemi OJ Helgerud J. Soccer specific aerobic endurance training. Br J Sports Med. (2002) 36(3):218–21. Available at: http://www.ncbi.nlm.nih.gov/pubmed/12055120 10.1136/bjsm.36.3.218 12055120 PMC1724499

[2] Bradley PS Carling C Gomez Diaz A Hood P Barnes C Ade J Match performance and physical capacity of players in the top three competitive standards of English professional soccer. Hum Mov Sci. (2013) 32(4):808–21. Available at: https://linkinghub.elsevier.com/retrieve/pii/S0167945713000687 10.1016/j.humov.2013.06.002 23978417

[3] Gualtieri A Rampinini E Sassi R Beato M. Workload monitoring in top-level soccer players during congested fixture periods. Int J Sports Med. (2020) 41(10):677–81. 10.1055/a-1171-1865 32455455

[4] Impellizzeri FM Marcora SM Coutts AJ. Internal and external training load: 15 years on. Int J Sports Physiol Perform. (2019) 14(2):270–3. 10.1123/ijspp.2018-0935 30614348

[5] Christopher J Beato M Hulton AT. Manipulation of exercise to rest ratio within set duration on physical and technical outcomes during small-sided games in elite youth soccer players. Hum Mov Sci. (2016) 48:1–6. 10.1016/j.humov.2016.03.013 27082027

[6] Kelly DM Gregson W Reilly T Drust B. The development of a soccer-specific training drill for elite-level players. J Strength Cond Res. (2013) 27(4):938–43. Available at: http://nsca.allenpress.com/nscaonline/?request=get-abstract&doi=10.1519%2FR-17254.1 10.1519/JSC.0b013e3182610b7d 22692111

[7] Hill-Haas SV Dawson B Impellizzeri FM Coutts AJ. Physiology of small-sided games training in football. Sport Med. (2011) 41(3):199–220. Available at: http://link.springer.com/10.2165/11539740-000000000-00000 10.2165/11539740-000000000-00000 21395363

[8] Dello Iacono A McLaren SJ Macpherson TW Beato M Weston M Unnithan VB Quantifying exposure and intra-individual reliability of high-speed and sprint running during sided-games training in soccer players: a systematic review and meta-analysis. Sport Med. (2023) 53(2):371–413. 10.1007/s40279-022-01773-1 PMC9877094 36331702

[9] Anderson L Orme P Di Michele R Close GL Milsom J Morgans R Quantification of seasonal-long physical load in soccer players with different starting status from the English premier league: implications for maintaining squad physical fitness. Int J Sports Physiol Perform. (2016) 11(8):1038–46. 10.1123/ijspp.2015-0672 26915393

[10] Beato M Coratella G Stiff A Dello Iacono A. The validity and between-unit variability of GNSS units (STATSports apex 10 and 18 hz) for measuring distance and peak speed in team sports. Front Physiol. (2018) 9(September):1288. 10.3389/fphys.2018.01288 30298015 PMC6161633

[11] Kelly DM Strudwick AJ Atkinson G Drust B Gregson W. Quantification of training and match-load distribution across a season in elite English premier league soccer players. Sci Med Footb. (2020) 4(1):59–67. 10.1080/24733938.2019.1651934

[12] Gualtieri A Rampinini E Dello Iacono A Beato M. High-speed running and sprinting in professional adult soccer: current thresholds definition, match demands and training strategies. A systematic review. Front Sport Act Living. (2023) 5:1116293. 10.3389/fspor.2023.1116293 PMC9968809 36860737

[13] Beato M Devereux G Stiff A. Validity and reliability of global positioning system units (STATSports viper) for measuring distance and peak speed in sports. J Strength Cond Res. (2018) 32(10):2831–7. Available at: http://www.ncbi.nlm.nih.gov/pubmed/30052603. 10.1519/JSC.0000000000002778 30052603

[14] Beato M De Keijzer KL Carty B Connor M. Monitoring fatigue during intermittent exercise with accelerometer-derived metrics. Front Physiol. (2019) 10(June):780. Available at: https://www.frontiersin.org/article/10.3389/fphys.2019.00780/full 10.3389/fphys.2019.00780 31293447 PMC6606691

[15] Silva H Nakamura FY Beato M Marcelino R. Acceleration and deceleration demands during training sessions in football: a systematic review. Sci Med Footb. (2022):1–16. 10.1080/24733938.2022.2090600. [Epub ahead of print] 35700979

[16] Impellizzeri FM Rampinini E Coutts AJ Sassi A Marcora SM. Use of RPE-based training load in soccer. Med Sci Sports Exerc. (2004) 36(6):1042–7. Available at: http://www.ncbi.nlm.nih.gov/pubmed/15179175 10.1249/01.MSS.0000128199.23901.2F 15179175

[17] Thorpe RT Strudwick AJ Buchheit M Atkinson G Drust B Gregson W. Monitoring fatigue during the in-season competitive phase in elite soccer players. Int J Sports Physiol Perform. (2015) 10(8):958–64. 10.1123/ijspp.2015-0004 25710257

[18] Scherr J Wolfarth B Christle JW Pressler A Wagenpfeil S Halle M. Associations between Borg's Rating of perceived exertion and physiological measures of exercise intensity. Eur J Appl Physiol. (2013) 113(1):147–55. 10.1007/s00421-012-2421-x 22615009

[19] Beato M Drust B Dello Iacono A. Implementing high-speed running and sprinting training in professional soccer. Int J Sports Med. (2021) 42(4):295–9. 10.1055/a-1302-7968 33291180

[20] Beato M. Recommendations for the design of randomized controlled trials in strength and conditioning. Common design and data interpretation. Front Sport Act Living. (2022) 4:981836. 10.3389/fspor.2022.981836 PMC9493045 36157898

[21] Beato M De Keijzer KL. The inter-unit and inter-model reliability of GNSS STATSports apex and viper units in measuring peak speed over 5, 10, 15, 20 and 30 meters. Biol Sport. (2019) 36(4):317–21. 10.5114/biolsport.2019.88754 31938002 PMC6945047

[22] Beato M Drust B. Acceleration intensity is an important contributor to the external and internal training load demands of repeated sprint exercises in soccer players. Res Sport Med. (2021) 29(1):67–76. 10.1080/15438627.2020.1743993 32200649

[23] Hopkins WG Marshall SW Batterham AM Hanin J. Progressive statistics for studies in sports medicine and exercise science. Med Sci Sports Exerc. (2009) 41(1):3–13. Available at: http://www.ncbi.nlm.nih.gov/pubmed/19092709 10.1249/MSS.0b013e31818cb278 19092709

[24] Stone NM Kilding AE. Aerobic conditioning for team sport athletes. Sports Med. (2009) 39(8):615–42. Available at: http://www.ncbi.nlm.nih.gov/pubmed/19769413 10.2165/00007256-200939080-00002 19769413

[25] Riboli A Coratella G Rampichini S Ce E Esposito F. Area per player in small-sided games to replicate the external load and estimated physiological match demands in elite soccer players. PLoS One. (2020) 15(9):e0229194. 10.1371/journal.pone.0229194 32966305 PMC7510966

[26] Duhig S Shield AJ Opar D Gabbett TJ Ferguson C Williams M. Effect of high-speed running on hamstring strain injury risk. Br J Sports Med. (2016) 50(24):1536–40. 10.1136/bjsports-2015-095679 27288515

[27] Vanrenterghem J Nedergaard NJ Robinson MA Drust B. Training load monitoring in team sports: a novel framework separating physiological and biomechanical load-adaptation pathways. Sports Med. (2017) 47(11):2135–42. Available at: http://www.ncbi.nlm.nih.gov/pubmed/28283992 10.1007/s40279-017-0714-2 28283992

[28] Fusco A Knutson C King C Mikat RP Porcari JP Cortis C Session RPE during prolonged exercise training. Int J Sports Physiol Perform. (2020) 15(2):292–4. Available at: https://journals.humankinetics.com/view/journals/ijspp/15/2/article-p292.xml 10.1123/ijspp.2019-0137 31172830

[29] Foster C Boullosa D McGuigan M Fusco A Cortis C Arney BE 25 Years of session rating of perceived exertion: historical perspective and development. Int J Sports Physiol Perform. (2021) 16(5):612–21. Available at: https://journals.humankinetics.com/view/journals/ijspp/16/5/article-p612.xml 10.1123/ijspp.2020-0599 33508782

[30] Iacono AD Unnithan V Shushan T King M Beato M. Training load responses to football game profile-based training (GPBT) formats: effects of locomotive demands manipulation. Biol Sport. (2022) 39(1):145–55. 10.5114/biolsport.2021.102919 35173373 PMC8805366

[31] Reynolds J Connor M Jamil M Beato M. Quantifying and comparing the match demands of U18, U23, and 1ST team English professional soccer players. Front Physiol. (2021) 12:706451. 10.3389/fphys.2021.706451 34276425 PMC8283180

[32] Stevens TGA de Ruiter CJ Twisk JWR Savelsbergh GJP Beek PJ. Quantification of in-season training load relative to match load in professional Dutch eredivisie football players. Sci Med Footb. (2017) 1(2):117–25. 10.1080/24733938.2017.1282163

[33] Kyprianou E Di Salvo V Lolli L Al Haddad H Villanueva AM Gregson W To measure peak velocity in soccer, let the players sprint. J Strength Cond Res. (2019) 36(1):273–276. 10.1519/JSC.0000000000003406 31800476

[34] Zamparo P Pavei G Nardello F Bartolini D Monte A Minetti AE. Mechanical work and efficiency of 5+5 m shuttle running. Eur J Appl Physiol. (2016) 116(10):1911–9. Available at: http://link.springer.com/10.1007/s00421-016-3443-6 10.1007/s00421-016-3443-6 27473448

[35] Zamparo P Zadro I Lazzer S Beato M Sepulcri L. Energetics of shuttle runs: the effects of distance and change of direction. Int J Sports Physiol Perform. (2014) 9(6):1033–9. 10.1123/ijspp.2013-0258 24700201

[36] Zamparo P Bolomini F Nardello F Beato M. Energetics (and kinematics) of short shuttle runs. Eur J Appl Physiol. (2015) 115(9):1985–94. 10.1007/s00421-015-3180-2 25963378

[37] Vigh-Larsen JF Dalgas U Andersen TB. Position-specific acceleration and deceleration profiles in elite youth and senior soccer players. J Strength Cond Res. (2018) 32(4):1114–22. Available at: https://journals.lww.com/00124278-201804000-00028 10.1519/JSC.0000000000001918 28699924

[38] Wass J Mernagh D Pollard B Stewart P Fox W Parmar N A comparison of match demands using ball-in-play versus whole match data in professional soccer players of the English championship. *Sports (Basel)*. (2021) 9(6):76. 10.3390/sports9060076 34073473 PMC8228731

[39] Okholm Kryger K Wang A Mehta R Impellizzeri FM Massey A McCall A. Research on women's Football: a scoping review. Sci Med Footb. (2021):1–10. Available at: https://www.tandfonline.com/doi/full/10.1080/24733938.2020.1868560 10.1080/24733938.2020.1868560 36540910

[40] Buchheit M Haddad HA Simpson BM Palazzi D Bourdon PC Salvo VD Monitoring accelerations with GPS in football: time to slow down? Int J Sports Physiol Perform. (2014) 9:442–5. 10.1123/ijspp.2013-0187 23916989

