# Supplementary material for: External and internal training load comparison between sided-game drills in professional soccer
Source: Front Sports Act Living. 2023 Apr 4;5:1150461. doi: 10.3389/fspor.2023.1150461 (PMC10110967; doi:10.3389/fspor.2023.1150461)
Supplement: Supplementary file 1 [file Table1.docx]

**Supplementary material**

***Distance per minute -post-hoc***

***
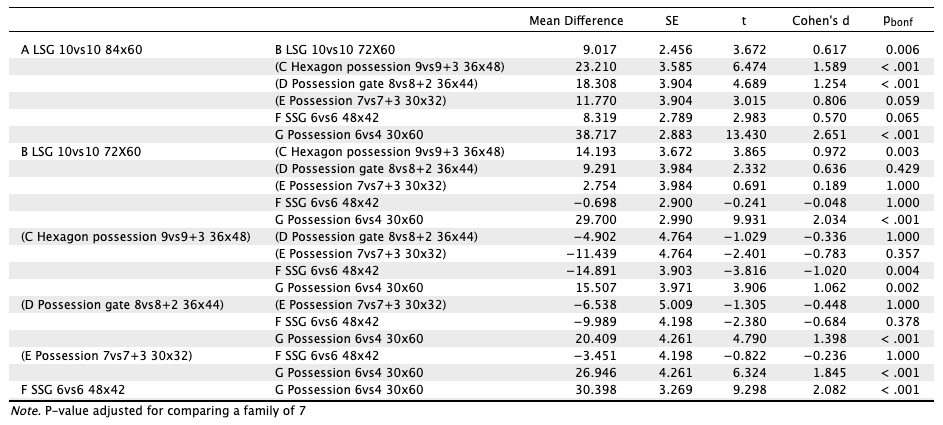
***

***HSR -post-hoc***

***
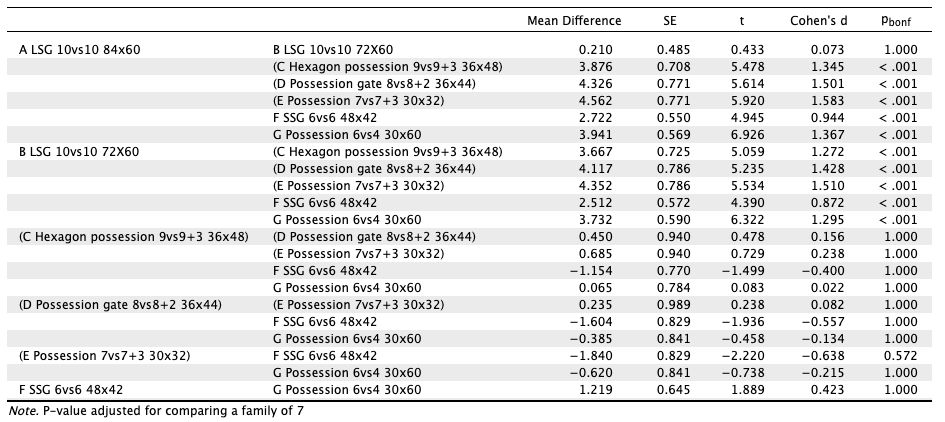
***

***Sprinting distance -post-hoc***

***
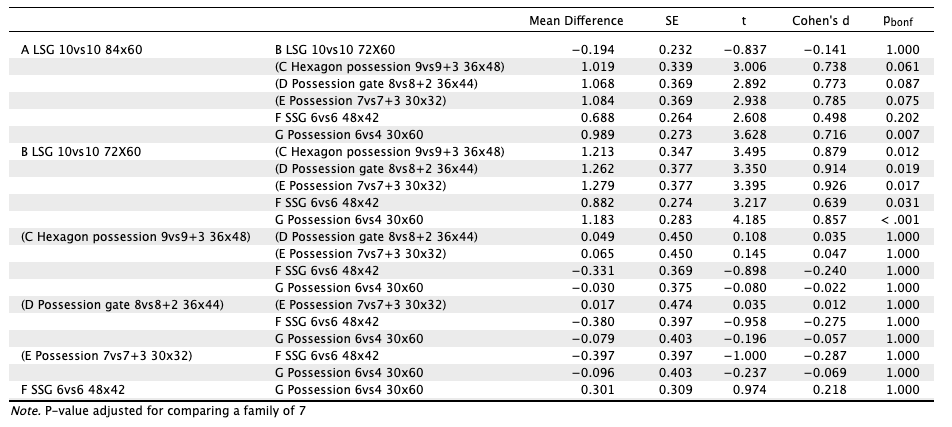
***

***Accelerations -post-hoc***

***
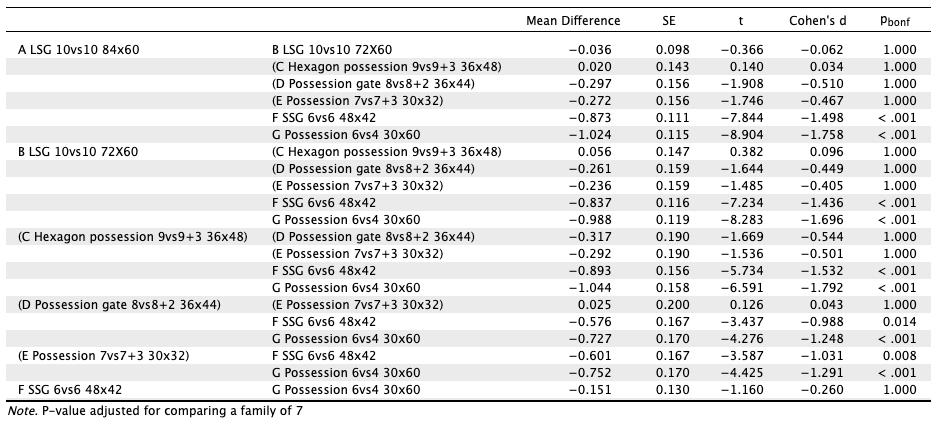
***

***Decelerations -post-hoc***

***
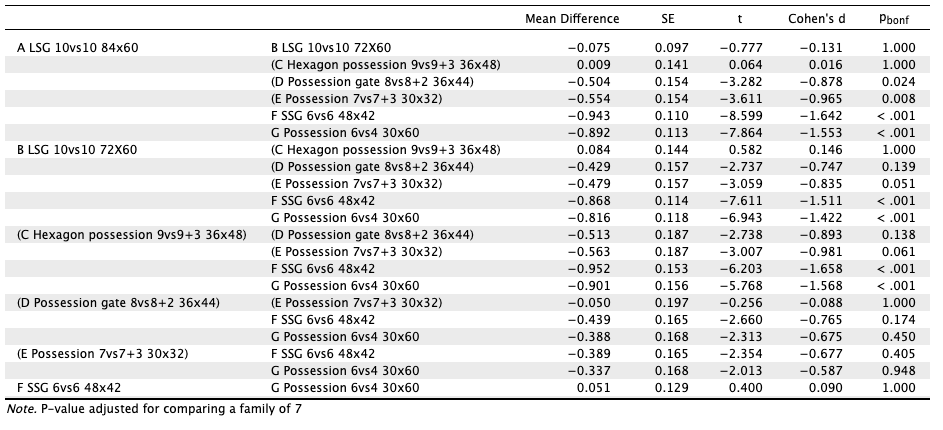
***

***RPE – post-hoc***

***
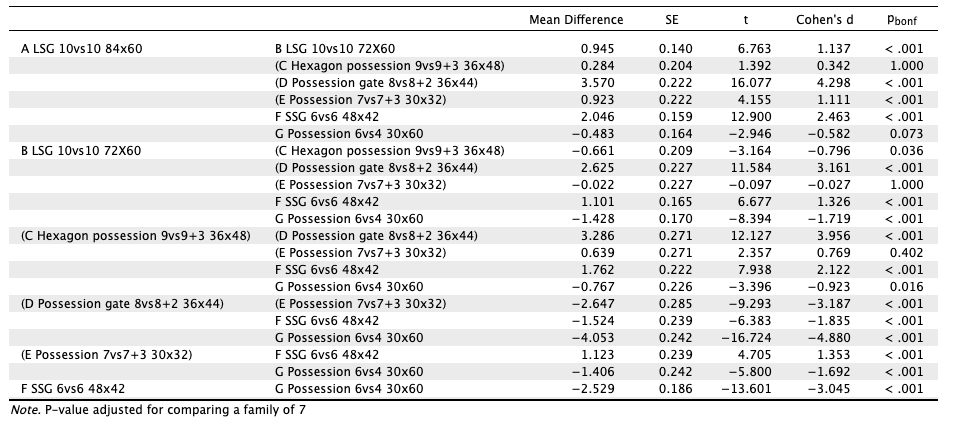
***
